# Evaluation of Cone-Penetration Test as a Rheology Quality-Control Field-Oriented Test for 3D Printing Cement-Based Systems

**DOI:** 10.3390/ma19051029

**Published:** 2026-03-07

**Authors:** Enrique Gomez, Hugo Varela, Gonzalo Barluenga

**Affiliations:** Departamento de Arquitectura, Universidad de Alcala, 28801 Madrid, Spain; enrique.gomezp@uah.es (E.G.); hugo.varela@uah.es (H.V.)

**Keywords:** 3D printing, extrusion, vane test, cone penetration test, squeeze test, cement pastes, shear and compressive yield stress

## Abstract

3D printing (3DP) of cement-based systems (CBSs) is a highly demanded technology in the construction field. Material requirements include specific rheological conditions for proper extrusion, followed by fast stiffening and strength gain to allow the construction process to continue, taking into account variable environmental conditions if the construction is on-site. To guarantee quality control of the process, it is essential to define field-oriented testing methodologies that allow real-time monitoring of mechanical properties’ evolution of the printed material, which will govern construction speed. This study evaluates the cone penetration test (CPT) method as a field-oriented test method to estimate the mechanical properties of 3DP CBSs over time. CPT penetration depth measurements were used to calculate shear yield stress and fresh compressive strength over time for 90 min. The experimental results were compared to two widely used laboratory tests: the fresh compressive strength test (squeeze test—SQT) and DSR test (vane test—VT). CBS pastes with and without fly ash and with three inorganic modifiers (nanoclays) and two types of organic rheology-modifying admixtures were considered. The results showed that CPT is highly conditioned by the stiffness of the paste, measured by the compressive Young Modulus (E), overestimating CBSs’ strength. The increase in E over time showed an inflection point at 130 kPa, corresponding to the evolution from plastic to pseudo-rigid behavior in the pastes. The corresponding time was used to define a linear adjustment for the average strength calculated using the CPT regarding both the fresh compressive SQT and shear yield stress VT.

## 1. Introduction

3D printing (3DP) with cement-based systems (CBS) has become a highly demanded and studied technology in the architectural construction sector [1,2,3]. However, there are still some issues that need further study in order to carry out an adequate and controlled implementation of the material’s rheology, extrudability and buildability [4,5,6].

During pumping and extrusion, 3DP CBSs require good cohesion to avoid segregation and water drainage [4,7]. In addition, after being 3D printed, the material requires an increase in strength over time in order to withstand the weight of the subsequent layer-by-layer structure, avoiding plastic deformation and buckling that would lead to collapse [8,9]. The main rheological parameters related to 3DP are shear yield stress (*τ*), compressive yield stress (*σ*) and the Young Modulus (*E*). These parameters must reach initial values within a printable interval (*τ*_0_ and *σ*_0_), while increasing over time, defining new time-dependent parameters such as effective thixotropy (*A_thix_*), compressive strength gain (Δ*σ*) and stiffening rate (*Ė*) [5,6,10,11]. The minimum initial stiffness can be achieved for *τ*_0_ above 1 kPa, while a minimum viscosity is required to reach initial cohesiveness [4,5,6].

The lack of sufficient fresh-state cohesion can be overcome by using rheology-modifying admixtures (RMAs), improving 3DP CBSs’ extrudability and buildability. RMAs can be organic- or inorganic-based CBS components. Cellulose-based or acrylamide-based organic RMAs are commonly used as viscosity-modifying admixtures (VMAs) [12,13,14], improving the operability of the 3DP process. Cellulose-based VMAs have proven to be useful to enhance extrudability but less effective for vertical constructability [6,12]. Among inorganic RMAs, nanoclays (NCs), such as sepiolite, attapulgite, bentonite and kaolin, can be used for 3DP CBSs [15,16,17,18,19]. NCs show high water and superplasticizer retention capacity due to colloidal attraction forces, which increase fresh mixture cohesion and effective thixotropy [6,17,20], something essential for CBSs’ constructability [2,21].

Although laboratory tests, such as DSR and fresh compressive strength, have a great accuracy and reproducibility in measuring rheological parameters, it is not possible to use them for on-site applications during the 3D printing process. Still, 3D printing technology needs real-time monitoring of a material’s strength and deformation evolution over time in order to optimize construction times based on process quality control. Consequently, there is an open debate in the 3D printing community to define reliable field-oriented tests of the rheological parameters of the fresh-state CBSs [1,22,23,24].

Several laboratory tests are commonly used, individually or combined, in 3DP with CBSs to compare their limits of use [6,24]. The main types are the extrusion test (or capillary rheometer) [6,25,26], slug test [27], slow penetration test [28,29], uniaxial fresh compression test or squeeze test (SQT) [28,30,31], and dynamic shear rheometer test (DSR) with different geometries, vane geometry being one of the most used in this field (vane test, VT) [6,22].

On the other hand, among the most used field-oriented tests in 3D printing are: the flow table test (FT) [32,33,34], the cone or cylinder slump test (CST) [32,34,35], the manual vane test (or pocket vane test) [36] and the fast cone penetration test [6,37,38]. These field-oriented experimental methods can be used in situ and can assess the mechanical behavior of the material [6,34,37].

The CPT is an easy and inexpensive test method that was originally used to measure the liquid limit and shear capacity of clay soils [39,40] and can also be applied to estimate CBSs’ rheological parameters [22] and to evaluate reversible and irreversible effective thixotropy (*A_thix_*) [6,34]. Initial shear (*τ*_0_) and compressive yield stress (*σ*_0_) can be calculated from CPT penetration depth [29,39,40], considering the von Mises plasticity criterion [28,38]. However, the transition from an elastic–plastic behavior into a granular pseudo-solid rigid material, produced by the physical–chemical bonds between water and cement, cannot be modeled accordingly and requires another approach [6].

In this study, experimental results of rheological parameters measured with two laboratory tests, the VT and SQT, were used to calibrate CPT-calculated values for CBSs in combination with three NCs and two VMAs. The aim of the study was to understand the limits and capabilities of the CPT as a reliable field-oriented test for the real-time monitorization of 3DP CBSs’ rheological parameters.

## 2. Experimental Program

### 2.1. Materials and Mix Design

A reference paste (R) with a cement type CEM I 52.5R and a w/b ratio of 0.27 was designed. Then, 25% of cement was replaced by fly ash (FA). The material’s consistency was adjusted using a polycarboxylate-ether-based superplasticizer, supplied by Master Builders Solutions España SL (Barcelona, Spain). These pastes were designed to reach an initial target CPT penetration depth of 20 ± 2 mm at 10 min. In addition, three types of nanoclay (NC) in powder form (2% with regard to cement weight) were incorporated to enhance the paste’s rheological properties:

- A bentonite clay with a platelet-shaped particle (C1), D(4,3)] of 38.42 μm, supplied by TOLSA Group (Madrid, Spain)
- A sepiolite clay with a needle-shaped particle (C2), D(4,3)] 39.66, supplied by TOLSA Group (Madrid, Spain)
- An attapulgite clay with a needle-shaped particle (C3), D(4,3)] 21.97 μm, supplied by TOLSA Group (Madrid, Spain).

Also, two viscosity modifier admixtures (VMAs) were used to improve mixture cohesion and extrudability:

- A polyacrylamide-co-acrylate-based VMA (V1), at 0.1% with regard to cement weight, Master Builder Solutions SL (Madrid, Spain).
- A cellulose ether-based VMA (V2), WALOCEL MKS 10000 PF 60, supplied by Dow Chemical Company (Midland, MI, USA) at 0.5% with regard to cement weight.

Table 1 summarizes the paste compositions proposed in this study [6]. Solid components were blended for one minute and the liquid components were added afterwards, reaching 5 min in total.

### 2.2. Experimental Methods and Test Procedures

**Figure 1:**
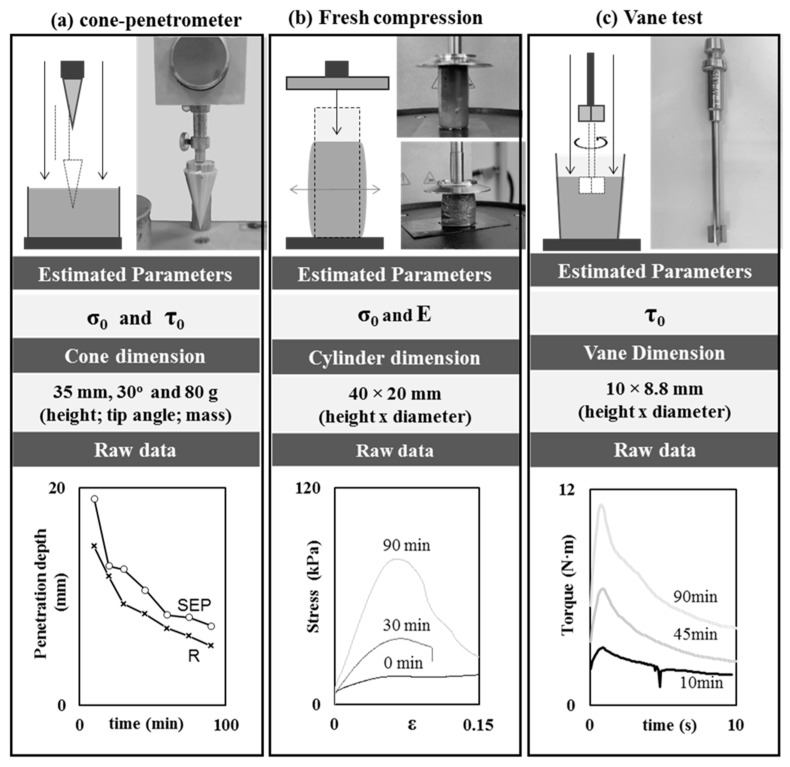
Experimental test methods used to evaluate rheological properties—main estimated parameters, main component dimensions and raw data obtained for: (**a**) cone penetration test (CPT); (**b**) fresh compression test (SQT); (**c**) vane test (VT).

Figure 1 describes the experimental test methods used in this study, as well as the main estimated rheological parameters, component or sample dimensions, and testing results. One field-oriented test, the cone penetration test (CPT), and two laboratory tests, the DSR vane test (VT) and a fresh compression test, also known as the squeeze test (SQT), were considered. The tests were performed on samples left to rest in time intervals between 10 and 90 min in order to analyze the evolution of their rheological and mechanical parameters over time.

**Figure 2:**
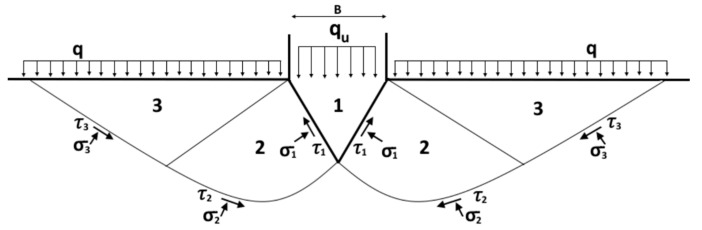
Failure model of a soil under the application of a conical load, from [41].

The cone penetration test (CPT) has traditionally been used in soil mechanics to calculate the shear failure point in foundation design. Shear yield strength (*τ*) is calculated at the plastic equilibrium point, according to the Prandtl-wedge failure mechanism (Figure 2) [41].

**Figure 3:**
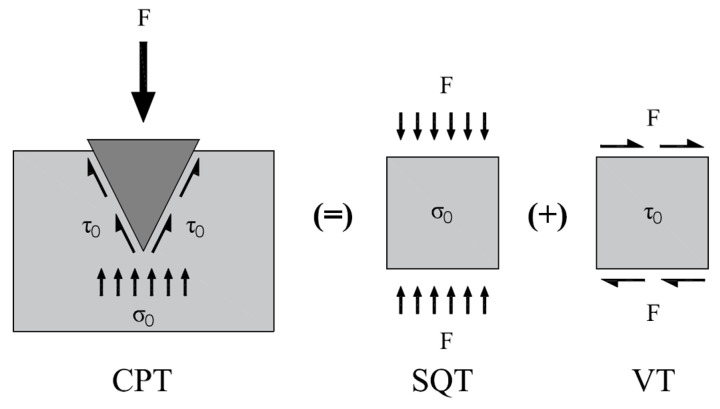
Stress diagram relating to the test methods used in the study.

Unlike soils with plastic behavior, cement-based systems (CBSs) for 3D printing applications stiffen and increase strength over time, due to physical–chemical bonds produced by cement hydration, transiting from elastic–viscous–plastic into pseudo-rigid behavior. Due to the penetration geometry and the mechanical evolution of the material over time, the CPT penetration depth values can be used to calculate shear (*τ*) and compressive (*σ*) strength (Figure 3).

The DSR vane test (VT) is a laboratory test method used to measure yield shear strength [6,11]. As shown in Figure 1, the test consists of inserting a vane-shape plunger with four blades into a sample and then applying a rotational force. The squeeze test (SQT) is a laboratory test that measures direct compressive strength on fresh paste samples, showing an initial elastic response followed by a plastic yielding stage [28,30,31].

#### 2.2.1. Cone Penetration Test (CPT)

The CPT was carried out with a digital soil penetrometer on 420 cm^3^ cement paste samples. The cone was 35 mm in height, with a 30° of tip angle (*θ*) and 80 g of mass (*m*) (Figure 1). Three to five penetrations were performed on each sample at each time to calculate *τ* and *σ* according to Equations (1) and (2), respectively [12]:

(1) $$\tau_{CPT}=\frac{m\;\times \;g\;\times \;{cos}^{2}(\frac{\theta }{2})}{\pi \times h^{2}\times \tan\left(\frac{\theta }{2}\right)}\;,$$

(2) $$\sigma_{CPT}=\frac{m\times g}{\pi \times h^{2}\times tg^{2}(\frac{\theta }{2})}\;,$$

where *m* is the cone mass, *g* is gravity (9.8 m/s^2^), *θ* is the cone tip and *h* is the average penetration height at each time (mm).

#### 2.2.2. Shear Strength: Vane Test (VT)

The VT was performed with an Anton Paar MCR 702 DSR (Anton Paar GmbH, Graz, Austria) equipped with a four blade-vane geometry (ST10-4V-8.8) on fresh paste samples at a constant rate of 0.05 s^−1^. Two plastic containers with 200 g of paste were employed to carry out the VT. Torque (*T_max_*) over time was obtained and *τ* was calculated using Equation (3) [42], considering the vane dimensions, 10 mm height (*H*) and 8.8 mm diameter (*D*):

(3) $$\tau_{VT}=\frac{T_{max}}{2\cdot \pi \cdot D^{2}\cdot (H+\left(\frac{D}{3}\right))\;}\;,$$

#### 2.2.3. Compressive Strength and Young Modulus: Squeeze Test (SQT)

The SQT was carried out with an Anton Paar MCR 702 DSR with a plate geometry at a constant velocity of 0.1 mm/s. Cylindric specimens of fresh paste with dimensions 20 mm × 40 mm, diameter × height (Figure 1), were cast in a plastic mold and demolded before being tested at different time intervals, and σ was calculated according to Equation (4):

(4) $$\sigma_{SQT}=\frac{F_{n}}{\left(\pi \cdot \;{\left(\frac{D}{2}\right)}^{2}\right)}\;,$$

where *Fn* was the axial force (N) and *D* the cylinder diameter (mm). The effective Young Modulus of pastes (*E*) was also measured with the SQT, considering the slope of the linear part of the stress–strain compression test curve [6].

## 3. Experimental Results

**Figure 4:**
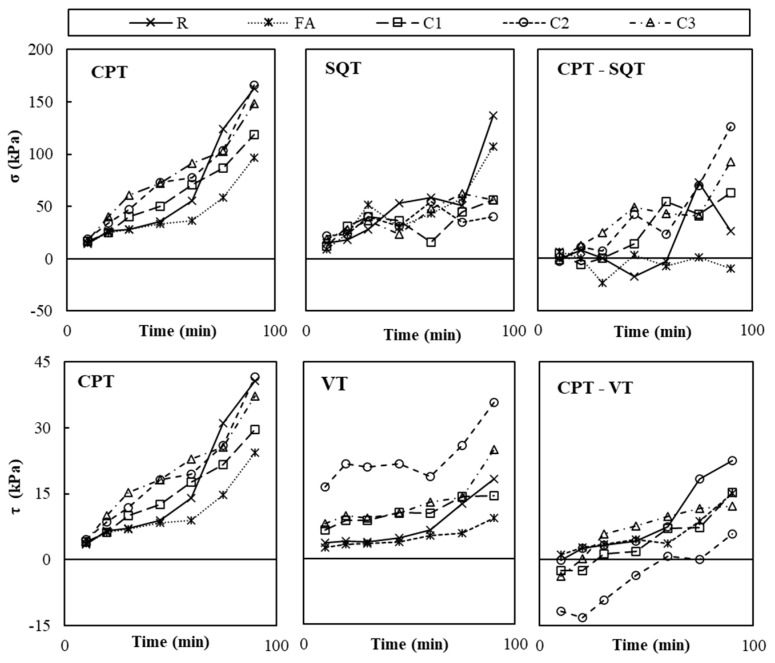
Experimental results of CPT, SQT and VTs of the WV pastes over time and difference between CPT values and SQT and VTs.

**Figure 5:**
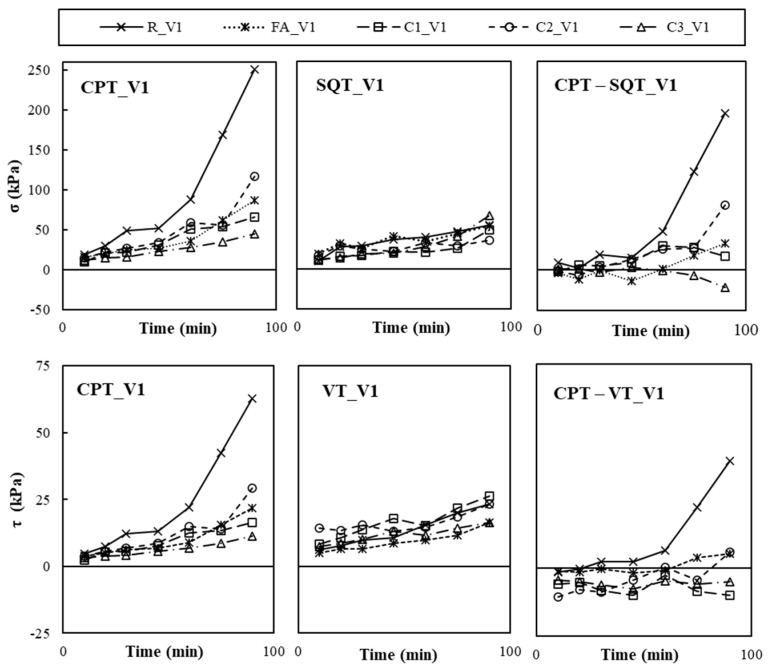
Experimental results of CPT, SQT and VTs of the V1 pastes over time and difference between CPT values and SQT and VTs.

**Figure 6:**
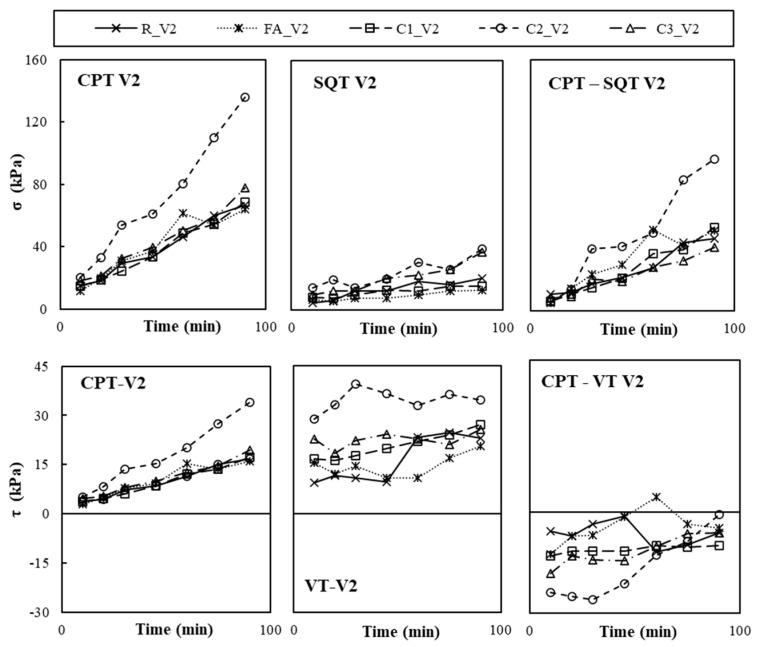
Experimental results of CPT, SQT and VTs of the V2 pastes over time and difference between CPT values and SQT and VTs.

The CPT, SQT, and VT were used on paste samples with different combinations of nanoclays and VMAs to calculate shear yield stress (*τ*), compressive yield stress (*σ*) and the Young Modulus (*E*). Figure 4, Figure 5 and Figure 6 plot the calculated values of *σ*, measured with the CPT and SQT, and *τ*, measured with the CPT and VT, of pastes without VMAs (Figure 4), with VMA type V1 (Figure 5) and with VMA type V2 (Figure 6).

It can be observed that the values of *σ* and *τ* increased over time in all cases and were primarily affected by the type of VMA and secondarily by the test type and the type of NC incorporated [6]. Values obtained with the CPT (*σ_CPT_* and *τ_CPT_*) were more accurate initially than the values obtained over time, when compared to the SQT (*σ_CPT_*) and VT (*τ_VT_*), respectively, and the difference grew larger with time, especially for *σ*.

**Figure 7:**
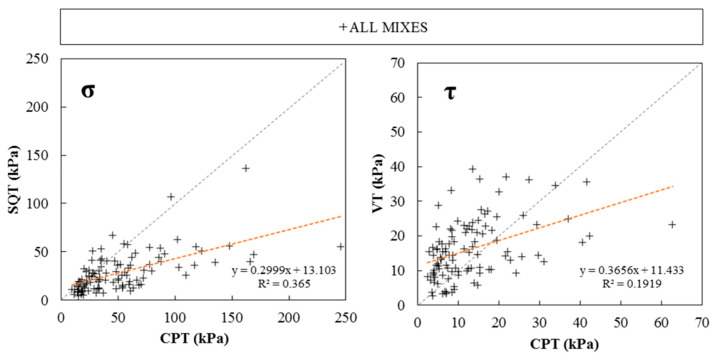
Experimental σ and τ results of CPTs and SQT and VTs.

Figure 7 compares *σ* and *τ* experimental results obtained with the CPT, SQT and VT. In both cases, results showed a poor correlation level (R^2^ of 0.36 and 0.19, respectively), pointing out a low predictability of material rheology and mechanical fresh properties from CPT (field-oriented) calculated using Equations (1) and (2).

## 4. Analysis and Discussion

### 4.1. Linear Analysis of τ and σ Average Values over Time

Table 2 summarizes *τ*, *σ* and *E* average values over time considering the type of VMA incorporated in the mixtures. The standard deviation of experimental results obtained for *σ* and *τ*, with the CPT, and SQT and VT, respectively, were calculated.

Regarding compressive strength, the WV and V1 mixtures showed similar σ up to 45 min from both tests, the SQT and CPT. Afterwards, *σ_CPT_* increased significantly regarding *σ_SQT_*. Pastes with V2 showed larger *σ_CPT_* than *σ_SQT_* since the initial values.

When yield shear strength (*τ*) was considered, it was observed that *τ_CPT_* underestimated yield values compared to *τ_VT_*, although the difference changed from positive to negative values in WV and V1 pastes, after 30 and 75 min respectively, which did not happen in the V2 paste. In addition, the standard deviation values were larger for *σ* than for *τ* (Table 2).

**Figure 8:**
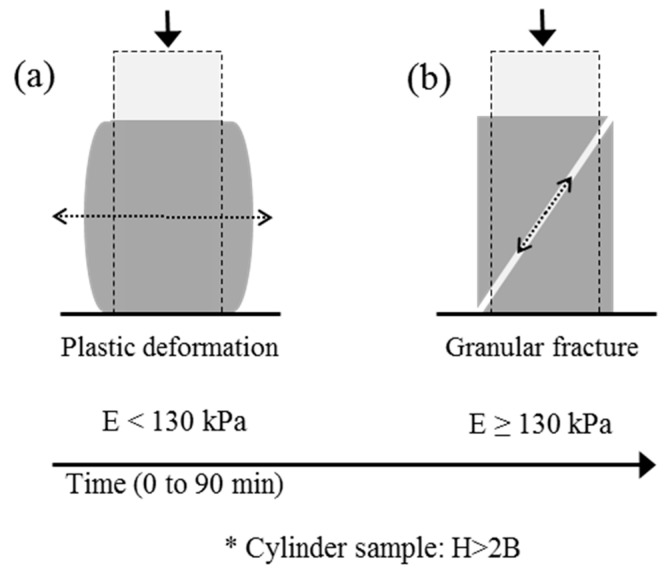
Evolution of the type of fracture of the pastes tested by SQT as a function of time, before and after reaching 130 kPa in the elasticity modulus.

The results of the average modulus of elasticity (*E*) showed a change in the stiffening ratio (*Ė*) of pastes without VMA and with V1 at 45 min (152.5 MPa and 128.4 MPa), while V2 pastes showed a constant stiffening ratio (linear increase) along the testing period. The change on *Ė* corresponded to the transition between plastic deformation and granular fracture observed in the SQT [6,15], identifying an E value of 130 kPa for WV and V1 pastes. Figure 8 explains the fracture patterns observed in the SQT of both plastic and granular deformation mechanisms, the threshold of which corresponded to an E value of 130 kPa [6,15].

**Figure 9:**
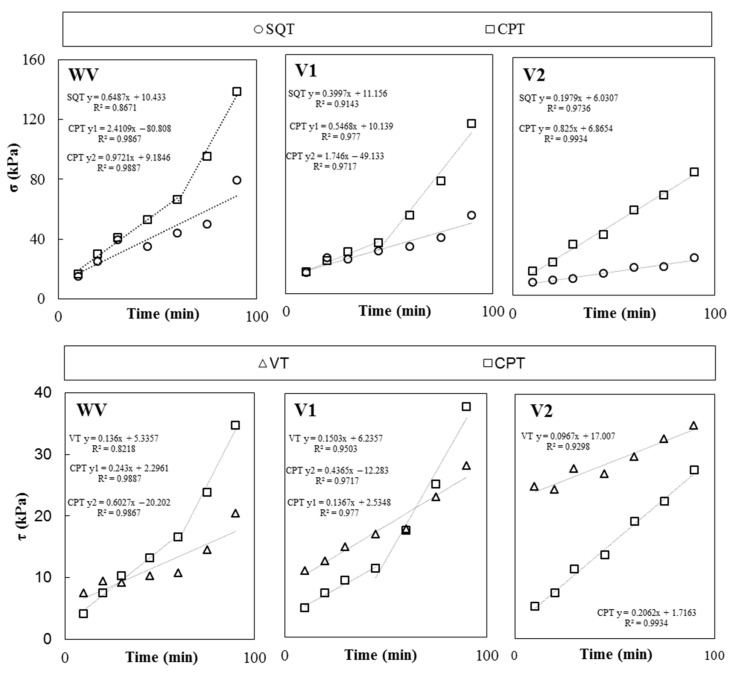
Average results of the CPT, SQT and VTs of the WV, V1 and V2 pastes.

Figure 9 plots the average σ and τ values computed on each test (SQT vs. CPT and VT vs. CPT, respectively) over time for pastes without VMAs (WV), with the polyacrylamide-co-acrylate-based VMA (V1) and with the cellulose ether-based VMA (V2).

Compressive strength gain (Δ*σ*) is the parameter that explains the variation in σ over time and can be calculated as the slope of a linear correlation of σ over time [5,6,10,11]. *σ_SQT_* evolution over time can be defined with one Δ*σ_SQT_* value up to 90 min for all pastes, independent of the type of VMA (Figure 9).

However, *σ_CPT_* requires consideration of the different behavior of the pastes before and after the transition from plastic to pseudo-rigid behavior due to physical–chemical particle interaction over time [5,6,9,11,13]. Accordingly, a bilinear correlation can be established considering the limit value of 130 kPa measured for E in SQT. It can be pointed out that the bilinear adjustment was only applied for VW and V1 pastes, while the V2 paste did not reach 130 kPa during the test (90 min).

Effective thixotropy (*A_thix_*) is the parameter that explains the variation in τ over time and can be calculated as the slope of a linear correlation of τ over time [5,6,10,11]. In this case, *τ_VT_* showed a good linear correlation over time up to 90 min (*A_thix_*_,*VT*_), while CPT required a bilinear correlation for ESQT values lower and higher than 130 kPa, which corresponded to τCPT of 12 kPa. Again, the bilinear adjustment only applied to the VW and V1 pastes.

### 4.2. Calibration Models of σ_CPT_ and τ_CPT_ Average Values

**Figure 10:**
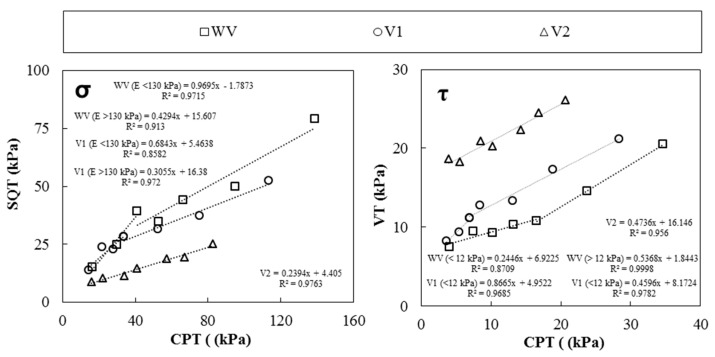
Linear modeling of the average experimental values obtained for CPT, and SQT and VT, for WV, V1 and V2 pastes.

Figure 10 compares σ and τ average values measured with the CPT (field-oriented test) and SQT and VT (laboratory tests), allowing the definition of calibration models. Linear correlations with a coefficient of determination (R^2^) higher than 0.86 can be established for each type of VMA used, due to the linear increase in both parameters over time (Δ*σ* and *A_thix_*) analyzed in Figure 9 [5,6,10,11].

Table 3 summarizes the linear equations obtained to calculate compressive strength (*σ_CAL_*) over time from *σ_CPT_* and (R^2^) when correlated to *σ_SQT_*. The change in Δ*σ* identified when E reached 130 kPa was considered for WV and V1 values, defining two intervals and, consequently, two calibration models. As V2 did not reach 130 kPa, one linear model was proposed.

On the other hand, Table 4 summarizes the linear equations obtained to calculate shear yield stress (*τ_CAL_*) over time from *τ_CPT_* and (R^2^) when correlated to *τ_VT_*. The change in *A_thix_* identified when *E* reached 130 kPa, which corresponded to *τ_CPT_* of 12 kPa, was considered for WV and V1 values, defining two intervals and, consequently, two calibration models. As V2 did not reach 130 kPa for *E*, one linear model was proposed.

### 4.3. Calibration Models’ Validation

**Figure 11:**
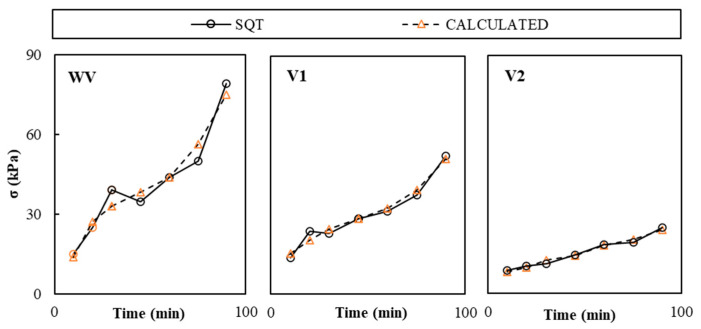
Experimental average SQT and calculated CPT compressive strength results.

Figure 11 compares the average compressive strength calculated from the CPT experimental results and calibration modes presented in Table 3 (*σ_CAL_*) and experimentally measured by SQT (*σ_SQT_*). The variability analysis of results is reported in Table 5 with values below 15.4%, which corresponded to VW pastes.

**Figure 12:**
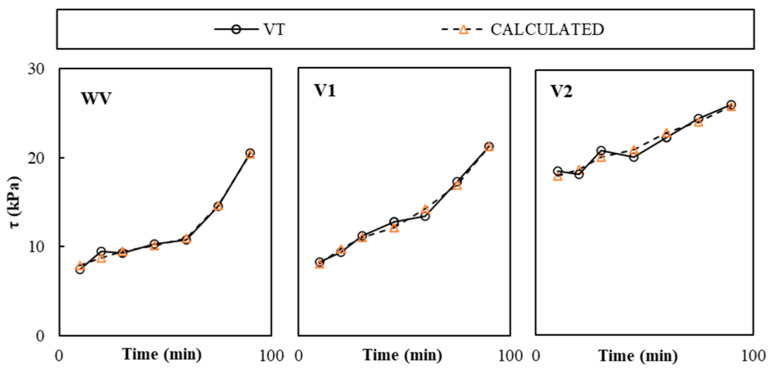
Experimental average VT and calculated CPT shear yield stress results.

Figure 12 compares the average shear yield strength calculated form CPT experimental results and calibration modes presented in Table 4 (*τ_CAL_*) and experimentally measured by VT (*σ_VT_*). The variability analyses of results are reported in Table 5 with values below 7.7%, which corresponded to VW pastes.

**Figure 13:**
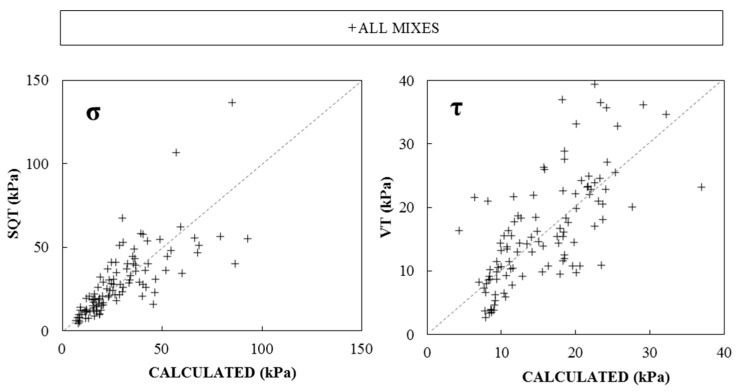
Calculated results of Compressive strength SQT and VT from the equations obtained with E modulus 130 kPa in SQT and Shear strength at 12 kPa in VT of all mixes.

Figure 13 plots the experimental and calculated results of σ and τ for all paste compositions with different combinations of VMAs and NCs. A strong reduction in the scattering between experimental and calculated values can be observed when compared to experimental results plotted in Figure 7. *σ_CAL_* showed lower differences than *τ_CAL_*, especially at lower values related to earlier stages, when paste behavior followed a plastic deformation mechanism and the penetration depths were deeper [6]. For later stages, when paste behavior followed a pseudo-rigid fracture, the dispersion was larger, due to the lower sensitivity when the CPT measured low penetration depths (approximately ≤ 4 mm depth).

Table 6 presents the experimental values of *σ_SQT_* and an analysis of the differences between *σ_SQT_* and *σ_CAL_* considering the different types of VMA and NC. The WV and V1 pastes showed larger differences than V2, reaching average values of 8.6 and 8.4 kPa in the first case and 3.7 kPa in the latter. In addition, the mean difference and standard deviation were larger at later times, due to the transition from plastic to pseudo-solid behavior [5,6,10,11]. The effect of NCs could not be tuned with the linear modeling as it depended on the type of VMA, which primarily governed fresh properties.

Table 7 presents the experimental values of *τ_VT_* and an analysis of the differences between *τ_VT_* and *τ_CAL_* considering the different types of VMA and NC. The WV and V2 pastes showed larger differences than V1, reaching average values of 6.9 and 6.2 kPa in the first case and 3.4 kPa in the latter. The mean difference and standard deviation remained quite constant in time and was not so affected by the plastic-to-rigid transition.

Accordingly, it can be said that the linear model proposed requires an experimental calibration for each RMA and can be used for any other modifier considered, as the model can be applied for the whole plastic-to-solid transition of cement-based mixtures.

## 5. Conclusions

The experimental values of compressive strength (σ) and shear yield stress (τ) in fresh cement-based systems (CBSs) for 3D printing applications with two types of viscosity modifying admixtures (VMAs) and three types of nanoclays (NCs) were analyzed. The results obtained by a field-oriented test, the cone penetrometer test (CPT), and two more precise laboratory tests, the compressive strength test, also known as the squeeze test (SQT) and DSR vane test (VT), were compared. Several linear calibration models were proposed to adjust the CPT experimental results in order to use the CPT as a quality control field-oriented test. The main findings of the study were:

- Laboratory tests SQT and VT were reliable testing methods to measure compressive and shear yield stress values of CBSs for 3D printing, as their measurements were not affected by the plastic-to-solid transition. However, they cannot be used as quality control tests.
- The CPT is a cheap field-oriented test method that can be used for the on-site measuring of compressive and shear mechanical properties over time for CBSs in 3D printing applications. However, calibration models are required to adjust the experimental values due to the influence of the plastic-to-solid transition of the fresh material.
- The type of VMA had a primary effect on the fresh properties evaluated. The polyacrylamide-co-acrylate-based VMA required a bilinear adjustment due to the transition from plastic to rigid behavior over time. On the contrary, the cellulose ether-based VMA delayed the transition and could be calibrated with a linear model.
- The transition from plastic to solid behavior was identified on pastes when the compressive Young Modulus (E) reached 130 kPa.
- The calibration models were defined considering the average σ and τ of mixtures with and without a VMA over time, producing calculated values from CPT measurements with reduced differences when compared to the laboratory tests’ results.

## Figures and Tables

**Table 1:** Pastes’ compositions.

Mixture	Cement (g)	Water (g)	Fly Ash (g)	HRWRA(%) *	C1 (g)	C2 (g)	C3 (g)	VMA1 (g)	VMA2 (g)
R	772	208.5	-	0.38	-	-	-	-	-
R-V1	772	208.5	-	1.5	-	-	-	0.77	-
R-V2	772	208.5	-	1.5	-	-	-	-	3.86
FA	579	208.5	193	0.5	-	-	-	-	-
FA-V1	579	208.5	193	1.5	-	-	-	0.77	-
FA-V2	579	208.5	193	1.25	-	-	-	-	3.86
C1	579	208.5	193	2.5	15.44	-	-	-	-
C1-V1	579	208.5	193	3	15.44	-	-	0.77	-
C1-V2	579	208.5	193	2.8	15.44	-	-	-	3.86
C2	579	208.5	193	3.5	-	15.44	-	-	-
C2-V1	579	208.5	193	2	-	15.44	-	0.77	-
C2-V2	579	208.5	193	5	-	15.44	-	-	3.86
C3	579	208.5	193	2	-	-	15.44	-	-
C3-V1	579	208.5	193	2	-	-	15.44	0.77	-
C3-V2	579	208.5	193	3.5	-	-	15.44	-	3.86

* Percentage with regard to cement weight.

**Table 2:** Analysis of average results of SQT, VT and CPTs, difference and standard deviation (SD) of WV, V1 and V2 pastes.

Mixture	Time (min)	σ SQT (kPa)	σ CPT (kPa)	Difference (kPa)	E SQT (kPa)	τ VT (kPa)	τ CPT (kPa)	Difference (kPa)
WV	10	15.1	16.3	−1.2	97	7.5	4.1	3.4
	20	24.9	30	−5.1	107.2	9.5	7.5	2
	30	39.2	40.9	−1.7	136	9.2	10.2	−1
	45	34.7	52.8	−18.1	152.5	10.3	13.2	−2.9
	60	44	66.3	−22.3	273.4	10.8	16.6	−5.8
	75	50	95.2	−45.2	399.1	14.5	23.8	−9.3
	90	79.2	138.6	−59.4	732.9	20.5	34.6	−14.1
SD (kPa)		-	-	21.0	-	-	-	5.8
V1	10	13.8	14.5	−0.7	64.8	8.2	3.6	4.6
	20	23.7	21.8	1.9	86.9	9.4	5.5	3.9
	30	23	27.9	−4.9	92.2	11.2	7	4.2
	45	28.4	33.7	−5.3	128.4	12.7	8.4	4.3
	60	31.4	52.4	−21	190.6	13.3	13.1	0.2
	75	37.4	75.3	−37.9	237.5	17.3	18.8	−1.5
	90	52.3	113.4	−61.1	402.3	21.1	28.3	−7.2
SD (kPa)		-	-	21.7	-	-	-	4.1
V2	10	8.6	15.9	−7.3	34.4	18.6	4	14.6
	20	10.2	22.2	−12	38.8	18.3	5.5	12.8
	30	11.2	34.2	−23	42.4	20.9	8.5	12.4
	45	14.6	40.9	−26.3	71.4	20.2	10.2	10
	60	18.6	57.3	−38.7	108.2	22.3	14.3	8
	75	19.3	67.2	−47.9	110.6	24.5	16.8	7.7
	90	25	82.6	−57.6	112.5	26.1	20.7	5.4
SD (kPa)		-	-	17.1	-	-	-	3.0

**Table 3:** Calibration models of calculated fresh compressive strength (*σ_CAL_*) over time for pastes, considering VMA type and *E_SQT_*, and coefficient of determination (R^2^) when correlated to *σ_SQT_*.

Mixture	E_SQT_ (kPa)	σ_CAL_	R^2^
WV	<130	0.97 σ_CPT_ − 1.8	0.97
	≥130	0.43 σ_CPT_ + 15.6	0.91
V1	<130	0.68 σ_CPT_ + 5.5	0.86
	≥130	0.31 σ_CPT_ + 16.4	0.97
V2	<130	0.24 σ_CPT_ + 4.4	0.98

**Table 4:** Calibration models of calculated yield shear strength (*τ_CAL_*) over time for pastes considering VMA type and *τ_VT_*, and coefficient of determination (R^2^) when correlated to *τ_VT_*.

Mixture	τ_VT_ (kPa)	τ_CAL_	R^2^
WV	<12	0.2 τ_CPT_ + 6.9	0.87
	≥12	0.5 τ_CPT_ + 1.8	0.99
V1	<12	0.9 τ_CPT_ + 4.9	0.98
	≥12	0.5 τ_CPT_ + 8.2	0.97
V2	≥12	0.5 τ_CPT_ + 16.1	0.96

**Table 5:** Average *σ_CAL_* and *τ_CAL_* of mixtures with different types of VMA and variability regarding average measured *σ_SQT_* and *τ_VT_*, respectively.

Mixture	Time	σ_CAL_ (kPa)	Variability σ_SQT_ vs. σ_CAL_ (%)	τ_CAL_ (kPa)	Variability τ_VT_ vs. τ_CAL_ (%)
WV	10	14.0	−7.0	7.9	6.3
	20	27.3	9.5	8.8	−7.7
	30	33.2	−15.4	9.4	2.0
	45	38.3	10.3	10.2	−1.1
	60	44.1	0.1	11.0	1.8
	75	56.5	12.9	14.6	0.6
	90	75.1	−5.1	20.4	−0.2
V1	10	15.4	11.3	8.1	−1.7
	20	20.4	−13.9	9.7	3.2
	30	24.6	6.9	11.0	−1.5
	45	28.6	0.6	12.0	−5.2
	60	32.4	3.1	14.2	6.4
	75	39.4	5.4	16.8	−2.6
	90	51.0	−2.4	21.2	0.3
V2	10	8.2	−4.5	18.0	−3.2
	20	9.7	−5.2	18.8	2.9
	30	12.6	12.6	20.2	−3.3
	45	14.2	−2.7	21.0	3.9
	60	18.1	−2.7	22.9	2.6
	75	20.5	6.3	24.1	−1.7
	90	24.2	−3.2	25.9	−0.8

**Table 6:** Experimental *σ_SQT_* (kPa), differences between calculated and experimental values, mean value and standard deviation (SD) of pastes with different types of VMA and NC.

MIXTURE		R		FA		C1		C2		C3			
V TYPE	TIME	σ_SQT_	σ_SQT_-σ_CAL_	σ_SQT_	σ_SQT_-σ_CAL_	σ_SQT_	σ_SQT_-σ_CAL_	σ_SQT_	σ_SQT_-σ_CAL_	σ_SQT_	σ_SQT_-σ_CAL_	Mean Diff (kPa)	SD (kPa)
WV	10	14.9	−0.8	8.8	−7.5	11.4	−6.2	21.4	−2.3	18.8	0.4	3.4	3.4
	20	18.3	−9.1	24.1	−1.6	31.0	5.1	23.4	−6.8	27.8	−12.8	7.1	7.0
	30	27.9	−1.4	25.1	−3.8	40.3	7.4	40.3	4.4	36.3	−5.4	4.5	5.5
	45	53.0	22.2	30.3	−3.8	35.9	−1.1	31.1	−15.8	23.2	−23.5	13.3	17.4
	60	58.3	18.7	43.2	6.5	39.3	−6.6	54.6	5.6	48.0	−6.8	8.8	10.6
	75	51.0	−17.8	57.8	17.0	44.4	−8.4	34.4	−25.8	62.4	2.7	14.3	16.9
	90	136.5	-	106.9	-	55.7	−10.8	40.2	-	56.4	−23.0	-	-
Mean Diff (kPa)			11.7		6.7		6.5		10.1		10.7	8.6	
SD (kPa)			14.3		8.3		6.4		11.1		9.7		10.1
V1	10	10.1	−8.7	19.1	3.0	11.2	−0.8	17.2	1.3	11.5	−2.5	3.3	4.5
	20	27.8	1.7	32.3	12.9	15.4	−4.6	29.2	8.5	13.9	−2.0	6.0	7.3
	30	29.4	−9.4	25.1	2.6	16.9	−3.7	24.4	0.3	19.1	2.3	3.7	5.1
	45	37.2	4.8	41.1	16.3	21.9	−4.9	21.6	−7.4	20.2	−3.2	7.3	9.6
	60	40.0	−3.4	34.8	7.5	23.5	−16.7	33.3	−1.2	28.2	3.3	6.4	9.2
	75	47.0	−21.2	44.4	8.9	25.9	−16.4	28.7	−4.9	41.0	14.1	13.1	15.3
	90	55.1	-	53.9	10.9	48.8	12.4	36.3	−16.0	67.3	37.0	19.1	21.7
Mean Diff (kPa)			8.2		8.9		8.5		5.7		9.2	8.4	
SD (kPa)			8.5		4.6		9.2		7.1		13.4		10.4
V2	10	4.4	−3.6	6.4	−0.8	8.1	0.2	14.3	5.1	9.8	1.1	2.2	3.2
	20	6.2	−2.5	5.3	−3.8	7.9	−1.0	19.5	7.1	12.3	2.8	3.5	4.4
	30	12.0	0.6	7.6	−4.2	9.6	−0.6	14.5	−2.9	12.2	0.1	1.6	2.0
	45	12.1	−0.4	7.8	−5.5	12.9	0.5	19.6	0.7	20.6	6.8	2.8	4.4
	60	18.6	3.2	9.6	−9.5	12.2	−3.8	30.5	6.9	22.3	5.8	5.8	7.0
	75	16.3	−2.4	12.4	−4.9	15.5	−1.9	26.1	−4.6	26.0	7.7	4.3	5.2
	90	20.6	0.2	12.5	−7.1	15.4	−5.5	39.2	2.2	37.3	14.3	5.9	8.5
Mean Diff (kPa)			1.8		5.1		1.9		4.2		5.5	3.7	
SD (kPa)			2.1		2.5		2.0		4.3		4.5		4.9

**Table 7:** Experimental *τ_VT_* (kPa), differences between calculated and experimental values, mean value and standard deviation (SD) of pastes with different types of VMA and NC.

MIXTURE		R		FA		C1		C2		C3			
V TYPE	TIME	τ_VT_	τ_VT_-τ_CAL_	τ_VT_	τ_VT_-τ_CAL_	τ_VT_	τ_VT_-τ_CAL_	τ_VT_	τ_VT_-τ_CAL_	τ_VT_	τ_VT_-τ_CAL_	Mean Diff (kPa)	SD (kPa)
WV	10	3.7	−4.1	2.6	−5.2	6.6	−1.3	16.3	12.0	8.0	−8.0	6.1	7.0
	20	4.0	−4.6	3.4	−5.1	8.7	0.2	21.6	15.2	9.8	−9.4	6.9	8.5
	30	3.8	−4.8	3.5	−5.2	8.7	−0.7	21.0	12.8	9.3	−10.6	6.8	7.9
	45	4.7	−4.4	3.8	−5.1	10.6	0.7	21.7	10.1	10.4	−11.6	6.4	7.2
	60	6.5	−3.8	5.4	−3.8	10.4	−0.9	18.7	6.4	13.0	−14.1	5.8	6.6
	75	12.6	−5.9	5.9	−4.6	14.3	0.8	25.9	10.2	14.0	−15.6	7.4	8.5
	90	18.1	−5.5	9.2	−3.6	14.4	−3.3	35.7	11.5	24.9	−21.8	9.2	10.6
Mean Diff (kPa)			4.7		4.7		1.1		11.2		13.0	6.9	
SD (kPa)			0.7		0.6		1.3		2.5		4.3		8.0
V1	10	6.3	−2.9	9.2	0.8	8.2	1.2	14.4	4.5	7.3	−0.3	1.9	2.4
	20	7.8	−3.7	7.8	−5.5	10.6	1.1	13.4	2.7	8.6	0.3	2.7	3.1
	30	9.9	−5.6	9.6	−9.5	13.8	3.0	15.5	4.2	10.2	1.7	4.8	5.4
	45	10.8	−5.5	12.4	−4.9	17.8	6.0	13.0	0.9	13.3	3.3	4.1	4.5
	60	15.4	−2.9	12.5	−7.1	15.4	1.4	14.6	−0.4	11.5	0.5	2.5	3.1
	75	20.1	−7.5	8.1	0.2	21.9	7.5	18.4	3.7	14.4	1.9	4.2	5.0
	90	23.2	−13.8	7.9	−1.0	26.3	10.6	23.4	1.7	16.3	1.5	3.7	7.8
Mean Diff (kPa)			6.0		4.2		4.4		2.6		1.4	3.4	
SD (kPa)			3.5		3.7		3.5		1.7		1.1		4.5
V2	10	9.5	−8.5	15.4	−2.1	16.7	−1.2	28.8	10.3	22.6	4.3	5.3	6.3
	20	23.2	−23.5	12.0	−6.5	16.2	−2.2	33.2	13.1	18.3	−0.4	9.1	11.8
	30	48.0	−6.8	14.5	−5.3	17.7	−1.3	39.4	16.9	22.1	2.1	6.5	8.5
	45	62.4	2.7	10.8	−9.8	19.8	−0.3	36.5	13.1	24.2	3.4	5.9	7.4
	60	56.4	−23.0	10.9	−12.5	22.1	0.1	32.8	7.1	22.8	0.7	8.7	10.8
	75	19.1	3.0	17.0	−5.5	23.9	1.3	36.2	7.0	21.0	−2.1	3.8	4.3
	90	32.3	12.9	20.6	−3.2	27.2	2.9	34.6	2.4	25.5	0.1	4.3	5.4
Mean Diff (kPa)			11.5		6.4		1.3		10.0		1.9	6.2	
SD (kPa)			12.6		3.4		1.6		4.5		2.1		7.8

## Data Availability

The original contributions presented in this study are included in the article. Further inquiries can be directed to the corresponding author.
